# Cattle Schistosomiasis With Farmers′ Practice Regarding the Prevention and Control of Zoonotic Trematodes in Maya City, Eastern Ethiopia

**DOI:** 10.1155/japr/7602193

**Published:** 2025-12-05

**Authors:** Yihenew Getahun Ambaw

**Affiliations:** ^1^ College of Veterinary Medicine, Haramaya University, Dire Dawa, Ethiopia, haramaya.edu.et

**Keywords:** cattle schistosomiasis, Ethiopia, preventive practices, zoonotic trematodes

## Abstract

In developing countries like Ethiopia, zoonotic trematodes have a significant economic impact on the animal and public health sectors. To determine the prevalence of cattle schistosomiasis with farmers′ practices toward the prevention and control of zoonotic trematodes, a cross‐sectional study was conducted among 423 cattle and 120 farmers in Maya City between October 2023 and May 2024 using simple random sampling. Out of 423 cattle, 78 (18.44%, 95% CI 15.01–22.44) were positive for schistosomiasis. For crossbred cattle (OR: 0.32, 95% CI: 0.14–0.73), old age (OR: 3.64, 95% CI: 1.74–7.63), poor body condition (OR: 4.9, 95% CI: 1.36–17.73), and extensive management (OR: 4.71, 95% CI: 2.53–8.76) were statistically significant factors for the prevalence of cattle schistosomiasis; however, sex, subcity, and farming type had no significant association with the prevalence of the diseases. Even though all farmers have a latrine in their home, 64.2% of the respondents defecated openly in the bush or in and around water. Most (80.8%) farmers also eat raw fish and raw vegetables, and around half of them (45%) did not clean the feeding and watering troughs of their animals regularly. Cattle farmers′ overall level of positive practice was 12.5%. The respondents′ education level had a significant association with positive practice, but gender, farming experience, marital status, age group, income per month, and subcity had no significant association. Cattle farmers′ practice toward the prevention and control of zoonotic trematodes is very low in Maya City; therefore, to mitigate the burden of this disease in animals and humans at the same time, promoting a one health approach is encouraged in Eastern Ethiopia.

## 1. Introduction

In different agroecological areas of Ethiopia, trematode parasites in ruminants and their related snail vectors are the main pressing concern. Ethiopia′s diverse agroecological regions, which vary from lowlands to highlands, provide different suitable habitats for the presence and spread of trematode parasites and their snails. Trematode parasites have a significant impact on the health of farm animals, causing considerable economic loss and public health threats [1]. *Schistosoma bovis* is a trematode parasite of ruminants spread by freshwater snails in sub‐Saharan Africa. The disease causes bovine intestinal schistosomiasis, which results in chronic illness and substantial economic loss in agricultural sectors [2].

Trematode infections, such as fascioliasis and schistosomiasis, are zoonotic snail‐borne parasitic diseases that put animal and human health at risk, and they are the major cause of the socioeconomic losses of many countries. Both the intermediate hosts and transmitting vectors for these waterborne parasitic diseases are snails [3]. Those trematodal diseases are tremendously prevalent in animals across tropical regions and are documented as the most significant parasites influencing animal production and causing significant economic loss [4].

In the world, schistosomiasis is a neglected tropical disease of both veterinary and medical importance. The endeavors to eliminate schistosomiasis as a public health problem and break the transmission dynamics to alleviate the possible zoonotic risk posed by animal *Schistosoma* species through viable hybridization in sub‐Saharan Africa have been essentially unnoticed [5]. Trematode infection in human beings is also considered an emerging public health threat and is normally transmitted through contact with intermediate hosts and consumption of contaminated food [6].

Globally, around 779 million human beings are at risk of getting the neglected tropical disease called bilharzia (human schistosomiasis), and it impacts more than 250 million people. The disease is disseminated in 78 endemic countries, of which 51 have high to moderate transmission rates, especially in deprived societies that have limited access to clean water and poor sanitation [7]. In Ethiopia, 37.5 million people are at risk of infection, and nearly 5.01 million people have suffered from schistosomiasis [8].

Individuals who work in a certain occupational sector, such as abattoirs, irrigation, animal farming, healthcare, and housewives, are at higher risk of getting fascioliasis and schistosomiasis. Previous reports have demonstrated a relationship between certain parasites and their intermediate host snails in general; however, limited evidence has concentrated on the main importance of snails and the mechanisms and involvement of intermediate snail hosts in the complex life cycle of snail‐borne parasites [9].

Furthermore, to describe the geographical distributions of schistosomiasis and fascioliasis, the basic biology of snail‐borne parasitic diseases and their hosts is very crucial. Thus, to reduce the exposure of ruminants to trematodes, snail control is compulsory since their treatment is very challenging due to the development of anthelmintic resistance in ruminant trematode parasites [10].

In Ethiopia, the prevalence of cattle schistosomiasis has been reported in different parts of the country, such as in South Achefer District (9.6% [11] and 22.2% [12]), in Central Ethiopia (Asela, Hawassa, and Batu) (6.0% [1]), in Haramaya (21.3% [13]), in South Wollo and Oromia Zones (16.7% [14]), in Bahir Dar (21.1% [15] and 16.5% [16]), and a systematic review and meta‐analysis (24.0% [17]). The disease is also prevalent in African countries such as in Senegal (92.0% [5]), in Zimbabwe (4.5% [18]), in Kenya (16.9% [19]), and in Côte d′Ivoire (22.5% in abattoirs and 0.8% in the field [20]) and in Asian countries like in Bangladesh (47.5% [21]), Pakistan (15.0% [22]), and the Philippines (0.0%–50.0% [23]).

The aforementioned epidemiological surveys are a signal of consistent occurrence of the diseases, particularly in animals grazing in swampy areas and waterlogged regions. The presence of biotypes comfortable for the reproduction and development of intermediate hosts is closely associated with the existence of stagnant water and swampy grazing lands [17].

To prevent and control snail‐borne parasitic diseases in endemic areas like Ethiopia, an assessment of the level of animal farmers′ practices toward the prevention and control methods of trematode infection and prevalence of the diseases is required. The findings of this study may help professionals working in a one health approach to keep path of the problems of parasitic zoonosis at diverse levels. It also provides evidence in implementing prevention and control measures of waterborne zoonosis correlated to parasitic diseases and in designing surveillance or further research. Therefore, the objective of this study was to generate evidence about the epidemiology of schistosomiasis in cattle and animal farmers′ practices to prevent trematode infections that can be used in planning and designing interventions in Eastern Ethiopia, as the WHO targets to eliminate the disease as a public health threat by 2030.

## 2. Material and Methods

### 2.1. Study Area

**Figure 1:**
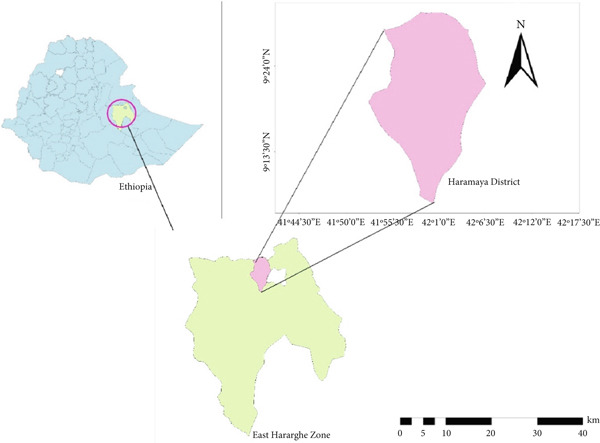
Map of Maya City, which is found in Haramaya District, East Hararghe Zone.

The study was conducted at Haramaya, Adele, and Awoday subcities in the Maya City of Haramaya District (Figure 1), Eastern Ethiopia, which is located 510 km to the east of Addis Ababa. The livestock population in the city was 120,350 caprine, 79,950 ovine, and 63,723 bovine. The city is topographically located at altitudes of 1600–2100 m above sea level, which places the study area into the category of highland. The city is found at 9°24 ^′^N 42°01 ^′^E at an altitude of 1950 m above sea level. The neighboring farming areas are semiarid. The average yearly estimated rainfall in the district varies from 600 to 1260 mm. The city is typical of a semihumid and midaltitude agroclimatic zone. The relative humidity ranges from 60% to 80%. The lowest yearly temperatures vary from 6°C to 12°C, whereas the highest vary from 17°C to 25°C [24].

### 2.2. Study Design and Study Population

A cross‐sectional study was carried out between October 2023 and May 2024 to assess the prevalence of cattle schistosomiasis and farmers′ practices toward the prevention and control of trematode infection in Maya City. The investigation incorporates both cross and local breed cattle older than 6 months of age. Cattle younger than 6 months of age, clinically ill, and that had been dewormed with praziquantel before 3 months from the beginning of the study were excluded from the study.

### 2.3. Sample Size Determination and Sampling Method

To determine the minimum required sample size, a single population proportion formula was applied [25]. Hence, using 5% absolute precision, a 95% confidence level, and a 50% expected prevalence provided a sample size of 384 study animals. The final sample size was adjusted by considering a 10% error rate in the laboratory procedure; the total sample size required becomes 423. Among the three subcities found in Maya City, 423 cattle were selected among 120 cattle farmers using a simple random sampling method by generating a random number in a Microsoft Excel spreadsheet 2013. The list of animal owners in those three subcities was obtained from the Maya City agricultural offices.

### 2.4. Fecal Examination in the Laboratory

The fecal sample of study animals was collected in the field directly from the rectum by wearing arm‐length disposable gloves. The compiled fecal sample was conserved by 10% formalin in clean and labeled screw‐capped universal bottles to impede the hatching of miracidia before arriving in the laboratory for investigation within 24 h after sample collection. Immediately after collection, the samples were put in an ice box and taken to Haramaya University, College of Veterinary Medicine, Parasitology Laboratory Room, for investigation. According to Hansen and Perry [26], the fecal samples were concentrated using the standard sedimentation method. In short, 3 g of feces was taken into a centrifuge tube and 40 mL of water was poured and mixed meticulously. The suspension was screened through a tea strainer into another centrifuge tube and was left for 15 min. Subsequently, the supernatant was decanted, and the sediment was resuspended. Until the supernatant was cleared, this procedure was repeated three times. Eventually, the sediment was conveyed with a Pasteur pipette on the clean microscopic slide and observed under a low‐power (10×) light microscope. The slides were considered to be positive if there was an oval to spindle‐shaped egg with a centrally bulged and terminal spine on one side of the egg detected.

### 2.5. Questionnaire Survey and Data Measurement Techniques

A pretested structured questionnaire was applied among 120 randomly selected cattle farmers to compile important information regarding the respondents′ practices for the prevention and control of trematodes in cattle after translation into a local language (Afan Oromic) using a face‐to‐face interview method. At the beginning of the interview, the objective of the research was explained in detail to each respondent, and an agreement from the participant was obtained. Each interview lasted approximately 15 min, and the interview had around 27 questions and two sections. The first section contains information regarding farmers′ sociodemographic factors (seven items), and the second section concerns the practice (20 items) of trematode prevention and control methods. The questionnaire was adopted from a previous study [27] and modified accordingly. The face and content validity of the tool were checked by a senior public health professional, parasitologist, and biostatistician, and some amendments were made based on their constructive feedback. The internal consistency of the questionnaire was evaluated by conducting a pilot study of 10 respondents who were omitted from the analysis. The outcome revealed a Cronbach′s alpha value of 0.84 for the practice domain, which discloses a good internal consistency questionnaire for respondents. The construct validity was also confirmed by using both the average variance extracted and the factor loading of the practice domain, with scores of 0.72, which was more than the usually recognized threshold level of 0.50, indicating good convergent validity of the constructs.

### 2.6. Data Management and Analysis

The collected data from both the questionnaire and laboratory analysis were documented and stored in a Microsoft Excel Spreadsheet Version 13. Both descriptive and inferential statistics were estimated by using Stata Version 16.0 software. The relationship between each covariate and the prevalence of cattle schistosomiasis was estimated by using crude odds ratios, and covariates that had a *p* value of less than 0.25 in the bivariate analysis were incorporated in the multivariable analysis. Thus, the independent variables such as breed, body condition, age group, subcities, and management practice were included in the multivariable model whereas sex and farming type were excluded from the final model. The presence of multicollinearity was verified by using the variance inflation factor; hence, management practice and watering point were highly correlated (multicollinear) with each other. As a result, the watering point was omitted from the multivariable model. The fitness of the final constructed multivariable binary logistic regression model was checked by using the Hosmer–Lemeshow test, and it was fitted for the cattle datasets with *χ* ^2^ test = 8.12, df = 8, and *p* = 0.422. For the questionnaire survey, cattle farmers had given a score of 1 if the question was answered correctly and 0 otherwise, for all practice questions. However, for all negative questions, the score was reversed. During data analysis, the mean score of respondents′ practice was used as the cut‐off point after adding all correct questions, and the study participants were again categorized into two groups. Each respondent who scored equal to the mean or more was categorized as having positive practice, while those respondents who scored lower than the mean were categorized as having negative practice [28]. The association of respondents′ practice with sociodemographic factors was also estimated by using the chi‐square test. During the whole data analysis stage, the authors used a 5% Type 1 error.

## 3. Result

**Figure 2:**
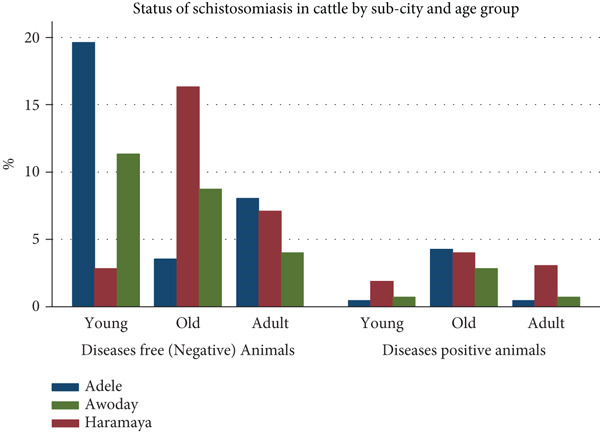
Status of cattle schistosomiasis by subcity and age group (*n* = 423) in Maya City, Eastern Ethiopia.

Four hundred twenty‐three cattle were selected randomly to determine the prevalence and associated risk factors of schistosomiasis in cattle in Maya City. Most of the sampled animals were taken in Adele subcity (154, 36.41%), followed by Haramaya (149, 35.22%). The majority of the study animals were male (225, 53.19%), and 335 (79.20%) were local breed cattle. The overall prevalence of cattle schistosomiasis was 78 out of 423 (18.44%:15.01%–22.44%) through coprological examination. The largest prevalence was found in Adele subcity (36.41%), while the lowest was in Awoday (28.37%) (Table 1). In general, a higher prevalence of schistosomiasis was recorded in older cattle and those from Haramaya subcity compared to young, adults, and those found in Adele and Awoday (Figure 2).

**Table 1:** The association of risk factors with the status of schistosomiasis in cattle by chi‐square test in Maya City, Eastern Ethiopia (*n* = 423).

**Factors**	**Categories**	**No. of examined (%)**	**No. of positive (%)**	**χ** ^2^ **test (df)**	**p** **value**
Sex	Male	225 (53.19)	37 (16.44)	1.27 (1)	0.259
	Female	198 (46.81)	41 (20.71)		

Breed	Cross	88 (20.80)	07 (7.95)	8.12 (1)	0.004
	Local	335 (79.20)	71 (21.19)		

Body condition	Good	269 (63.59)	34 (12.64)	17.97 (2)	< 0.001
	Medium	139 (32.86)	38 (27.34)		
	Poor	15 (3.55)	6 (3.55)		

Age group	Young	156 (36.88)	13 (8.33)	20.76 (2)	< 0.001
	Adult	99 (23.40)	18 (18.18)		
	Old	168 (39.72)	47 (27.98)		

Subcity	Awoday	120 (28.37)	18 (15.00)	7.65 (2)	0.022
	Haramaya	149 (35.22)	38 (25.50)		
	Adele	154 (36.41)	22 (14.29)		

Management practice	Extensive	226 (53.43)	62 (27.43)	26.10 (1)	< 0.001
	Semi‐intensive	197 (46.57)	16 (8.12)		

Watering point	Pond and lake	222 (52.48)	59 (26.58)	20.57 (1)	< 0.001
	Tap water	201 (47.52)	19 (9.45)		

Farming type	Cattle only	179 (42.32)	35 (19.55)	0.26 (1)	0.613
	Mixed	244 (57.68)	43 (17.62)		

Overall prevalence		423 (100)	78 (18.44)	95 CI (15.01–22.44)	

The chi‐square test (*χ* ^2^) analysis demonstrated that the presence of a significant association between the prevalence of schistosomiasis and the risk factors breed, body condition, age group, subcity, management practice, and watering point (*p* < 0.05), whereas farming type and sex were not (*p* > 0.05) (Table 1).

Four variables, such as poor and medium body condition, crossbreed, old age, and intensive management, were found statistically significant (*p* < 0.05) risk factors for cattle schistosomiasis in the final multivariable binary logistic regression model. The prevalence of cattle schistosomiasis had a significant association with different breeds of cattle. Bing cross‐breed cattle can reduce the prevalence of schistosomiasis by 68% compared to the local breed. Age was also a risk factor, and cattle found in the old age group were 4.2 times more likely to be infected by schistosomiasis relative to the young age group (AOR = 4.27, 95% CI: 2.21–8.27; *p* = 0.001). Body condition was also a predictor for schistosomiasis, and for medium body condition cattle, the odds of developing schistosomiasis were two times more likely compared to poor body condition (AOR = 2.03, 95% CI: 1.15–3.58; *p* = 0.015). For cattle with poor body condition scores, the odds of developing schistosomiasis were 4.90 times more likely compared to good body conditions (AOR = 4.90, 95% CI: 1.36–17.73; *p* = 0.015). The management system was also a risk factor for the prevalence of cattle schistosomiasis. The occurrence of developing schistosomiasis in the extensive management system was 4.71 times more likely compared to the semi‐intensive management system (AOR = 4.71, 95% CI: 2.53–8.76; *p* = 0.001) (Table 2).

**Table 2:** Both univariable and multivariable binary logistic regression analyses of schistosomiasis in cattle (*n* = 423) in Maya City, Eastern Ethiopia.

**Factors**	**Categories**	**COR (95% CI)**	**p** **value**	**AOR (95% CI)**	**p** **value**
Sex	Male	Ref			
	Female	1.33 (0.81–2.17)	0.260	—	—

Breed	Cross	0.32 (0.14–0.73)	0.006	0.26 (0.11–0.64)	0.003 ^∗^
	Local	Ref		Ref	

Body condition	Good	Ref		Ref	
	Medium	2.60 (1.54–4.37)	< 0.001	2.03 (1.15–3.58)	0.014 ^∗^
	Poor	4.61 (1.54–13.76)	0.006	4.90 (1.36–17.73	0.015 ^∗^

Age group	Young	Ref		Ref	—
	Adult	2.44 (1.14–5.25)	0.022	1.90 (0.83–4.37)	0.131
	Old	4.27 (2.21–8.27)	< 0.001	3.64 (1.74–7.63)	0.001 ^∗^

Subcity	Awoday	Ref		Ref	
	Haramaya	1.94 (1.04–3.61)	0.037	1.59 (0.79–3.21)	0.198
	Adele	0.95 (0.48–1.85)	0.868	1.10 (0.52–2.33)	0.799

Management practice	Extensive	4.28 (2.37–7.71)	< 0.001	4.71 (2.53–8.76)	< 0.001 ^∗^
	Semi‐intensive	Ref		Ref	

Farming type	Cattle only	1.14 (0.69–1.86)	0.613	—	—
	Mixed	Ref			

*Note:* Overall model fitness by Hosmer–Lemeshow *χ* ^2^ test with eight df = 8.12 and *p* value = 0.422. Asterisk ( ^∗^) denotes presence of statistical significance.

Abbreviations: *χ* ^2^, chi‐square test; AOR, adjusted odds ratio; COR, crude odds ratio; df, degree of freedom.

### 3.1. Practices of Respondents Toward the Prevention of Cattle Schistosomiasis

Less than one‐third (27.5%) of the respondents were isolated diseased from sick animals, and the remaining 72.5% did not practice this preventive measure. During the disposal of fecal and other discharges, only 4.2% of the respondents utilized gloves, and 11.7% of the study participants kept newly bought animals in quarantine for some time before mixing with other animals. Nearly half (48.3%) of the respondents had shared a house with animals, and 72.5% of the study participants allowed their animals to go in the common grazing areas. Most (55.8%) of the respondents had regularly cleaned the dung of animal facilities and stored the dung piles (60.8%) for a longer period. Respondents also reported that, out of the 60.8% stored dung piles, 61.6% were accessed by other animals. Around two‐thirds (65.0%) of the respondents had practiced slaughtering animals at livestock facilities, and 45.8% of participants tried to reduce snails from their animals′ environment. Most (90.8%) of the respondents had dewormed their animals with anthelmintics to prevent trematodes, and more than three‐fourths (78.3%) of the respondents had frequent contact with water. All the study participants (100%) had latrine facilities in their house, and around two‐thirds (64.2%) had practiced open defecation in the bush, in an open place, or in and around the water bodies. Overall, 15 (12.5%) of the respondents had positive practices toward the prevention and control of trematode infection, whereas 105 (87.5%) had negative practices (Table 3).

**Table 3:** Respondents′ practices to prevent the transmission of schistosomiasis among cattle in Maya City, Eastern Ethiopia (*n* = 120).

**Q no.**	**Practice-related questions**	**Categories**	**n**	**%**
Q_1_	Do you isolate diseased animals from healthy animals on your farm?	Yes	33	27.5
		No	87	72.5
Q_2_	Do you wear gloves during disposing of fecal and other discharges?	Yes	5	4.2
		No	115	95.8
Q_3_	Do you retain (keep) newly bought animals in quarantine for some time?	Yes	14	11.7
		No	106	88.3
Q_4_	Do you keep animals by mixing different species and ages?	Yes	89	74.2
		No	31	25.8
Q_5_	Do you use any type of protective clothing during handling animals?	Yes	41	34.2
		No	79	65.8
Q_6_	Do you allow your animals to go in the common grazing areas?	Yes	87	72.5
		No	33	27.5
Q_7_	Do you disinfect the shades where your animals are kept?	Yes	7	5.8
		No	113	94.2
Q_8_	Do you live in common places (houses) with animals?	Yes	58	48.3
		No	62	51.6
Q_9_	Do you clean the dung of your animal facilities?	Yes	67	55.8
		No	53	44.2
Q_10_	Do you store the dung piles for a longer period?	Yes	73	60.8
		No	47	39.2
Q_11_	Do other animals have access to these longer‐period stored dung piles?	Yes	45	61.6
		No	28	38.4
Q_12_	Do you slaughter animals at your animal facilities?	Yes	78	65.0
		No	42	35.0
Q_13_	Do you clean the feeding and watering troughs of animals?	Yes	66	55.0
		No	54	45.0
Q_14_	Do you practice reducing snails from your animals′ feeding and watering troughs?	Yes	55	45.8
		No	65	54.2
Q_15_	Do you swim or wash your body in a pond, river, deep well, stream, or lake?	Yes	41	34.2
		No	79	65.8
Q_16_	Do you deworm your animals with anthelmintics?	Yes	109	90.8
		No	11	9.2
Q_17_	Do you have a latrine facility in your house?	Yes	120	100.0
		No	0	0.0
Q_18_	Do you defecate openly in the bush, in an open place, or in and around the water?	Yes	77	64.2
		No	43	35.8
Q_19_	Do you eat raw fish meat and vegetables?	Yes	97	80.8
		No	23	19.2
Q_20_	Do you have frequent contact with open fresh water or lakes?	Yes	94	78.3
		No	26	21.7
	Total practice level	Positive	15	12.5
		Negative	105	87.5

### 3.2. Respondents′ Sociodemographic Characteristics

One hundred twenty smallholder cattle farmers were interviewed to generate evidence regarding the practices of prevention and control of trematode infection in Maya City, Eastern Ethiopia. Around 57.5% of the respondents were females, 82.5% were married, and 55.8% were aged between 31 and 68 years old. Regarding the educational status of the respondents, 14.2% had attained high school and above, 29.1% had followed elementary school, and 56.7% had not followed any formal education. The majority (37.5%) of respondents were from Adele, followed by Haramaya (35.8%), and most (60.0%) of the participants had more than 10 years of farming experience (Table 4).

**Table 4:** Association of respondents′ demographic variables with the level of practices toward the prevention of trematodes in Maya City, Eastern Ethiopia (*n* = 120).

**Demographic variables**	**Characteristics**	**N** **(%)**	**Total practice level**		**χ** ^2^ **test (df)**	**p** **value**
			**Positive (%)**	**Negative (%)**		
Gender	Male	51 (42.5)	6 (11.8)	45 (88.2)	0.04 (1)	0.834
	Female	69 (57.5)	9 (13.0)	60 (87.0)		

Marital status	Single	21 (17.5)	3 (14.3)	18 (85.7)	0.07 (1)	0.785
	Married	99 (82.5)	12 (12.1)	87 (87.9)		

Educational status	No formal education	68 (56.7)	4 (5.9)	64 (94.1)	7.86 (2)	0.020 ^∗^
	Elementary	35 (29.1)	6 (17.1)	29 (82.9)		
	High school and above	17 (14.2)	5 (29.4)	12 (70.6)		

Age group	18–30 years	53 (44.2)	7 (13.2)	46 (86.8)	0.04 (1)	0.835
	31–68 years	67 (55.8)	8 (11.9)	59 (88.1)		

Income per month	≤ 10,000 birr	44 (36.7)	5 (11.4)	39 (88.6)	0.08 (1)	0.775
	> 10,000 birr	76 (63.3)	10 (13.2)	66 (86.8)		

Subcities	Awoday	32 (26.7)	3 (9.4)	29 (90.6)	0.70 (2)	0.705
	Haramaya	43 (35.8)	5 (11.6)	38 (88.4)		
	Adele	45 (37.5)	7 (15.6)	38 (84.4)		

Farming experience	≤ 10 years	48 (40.0)	5 (10.4)	43 (89.6)	0.32 (1)	0.573
	> 10 years	72 (60.0)	10 (13.9)	62 (86.1)		

*Note:* Asterisk ( ^∗^) denotes presence of statistical significance.

Abbreviations: *χ* ^2^, chi‐square test; df, degree of freedom; *N*, sample size.

The result of the chi‐square test revealed that there was a significant association between the overall practice level and the educational status of the farmers (*p* = 0.020), whereas sex, marital status, subcities, income per month, farming experience, and age group had no association (*p* > 0.05) (Table 4).

## 4. Discussion

The objective of this study was to determine the prevalence of cattle schistosomiasis, associated factors, and farmers′ practices to prevent and control zoonotic trematodes. The current coprological examination disclosed that the total prevalence of schistosomiasis in cattle was 18.44% (95% CI: 15.01–22.44). This finding was in line with the previous reports in South Wollo and Oromia Zone (16.7% [14]), in and around Bahir Dar (16.5% [16]), and in and around Haramaya (21.28% [13]). However, the current finding was higher than earlier studies conducted in and around Nekemte (5.7% [29]), in Central Ethiopia (6.3% [1]), in South Achefer District (9.6% [11]), in Tis Abay District (13.02% [30]), in Côte d′Ivoire (0.7% [20]), and in Pakistan (15% [22]). This difference in the prevalence of schistosomiasis among cattle in diverse study areas might be attributed to the variation in the presence of suitable habitat for the snail intermediate hosts, seasons of the study, animal management practices, and agroecological conditions between the study areas [31, 32]. In addition, the availability of comfortable everlasting water bodies, wetness, differences in temperature, humidity, and irrigation techniques are essential supplementary elements that support the life cycle of *Schistosoma* and increase their abundance [13]. The higher prevalence in the current finding in Maya City could be owing to the existence of recognized water bodies (Haramaya lake) in the study area that may favor the multiplication and growth of the trematode parasite intermediate hosts (snail) and the practice of farmers to allow free grazing of their animals on the swampy and stagnant water pasture lands might be an additional predisposing risk factor for the diseases. In agreement with these ideas, authors [13, 14] describe the significance of swampy areas and water bodies for the prevalence of schistosomiasis. This issue was also supported by previous researchers, who stated that the occurrences and prevalence of schistosomiasis in a certain area might be affected by the presence of suitable intermediate and final hosts, local climate conditions, and the presence of water bodies, lakes, and rivers [33, 34].

On the other hand, this study was also lower than a systematic review and meta‐analysis in Ethiopia (24% [17]), in the Dembia District (25.9% [35] and 27.13% [36]), and in Bangladesh (47.5% [21]). The possible reason for this lower prevalence in the current study could be that the diagnostic test employed to detect the presence of *Schistosoma* eggs in the sampled animal was only the coprological test, which may have lower sensitivity to identify *Schistosoma* eggs in bovine feces. This idea is also supported by Bushara et al. [37], which states that trematode parasites are irregular egg layers, so that the likelihood of identifying eggs during a single fecal sample examination may be limited, and variation in sampling seasons might be another possible factor.

The present result demonstrated that the prevalence of schistosomiasis infection in cattle was significantly different among age groups (*p* = 0.001). This finding was comparable with previous studies in Ethiopia and across the world [11, 13, 14, 20, 30], which indicates that the prevalence of schistosomiasis was different between different age groups, and a higher prevalence was observed in adult and older animals. This condition might be attributed to the fact that cattle found in adult and old age groups cover large areas and have high grazing capacity relative to young age groups under extensive and semi‐intensive management systems, where the prevalence of cercariae infection is high [14]. Moreover, as the age of cattle escalates, the incidence of schistosomiasis may decline due to the development of immunity in chronically infected animals, which again decreases the production of parasite eggs. The immunity of animals not only inhibits the challenge of infection but also impedes worm fecundity, which leads to a decline in the fecal egg count. This is in opposition to the increase in worm burdens of schistosomiasis with the age of naturally infected animals. Besides, the duration of exposure to continuous schistosomiasis also decreases the susceptibility to reinfection in cattle [13].

The current study also revealed that the breed of cattle was significantly associated (*p* = 0.003) with the prevalence of schistosomiasis. The prevalence of schistosomiasis in crossbred cattle was lower than in local breed animals. The result was consistent with the former reports [13, 14]. The lower prevalence in crossbred cattle might be because crossbred cattle are kept indoors for dairy production in intensive or semi‐intensive management systems by feeding quality concentrate, roughages, and clean water, whereas local breed cattle were managed outdoors and repeatedly exposed to *Schistosoma* infection more than the crossbred animals [12].

In this investigation, the prevalence of schistosomiasis was significantly different (*p* < 0.05) between the body conditions of animals. The occurrence of the disease was higher in poor body condition cattle relative to medium and good body condition cattle. The result was in line with the existing findings [1, 12–14, 30], which indicate that the level of infection was greater in cattle with poor body condition scores. This higher prevalence in poor body condition cattle might be due to the compromised and susceptible state of immunity in poor body condition and weak animals, which becomes more suppressed and susceptible to the disease, possibly due to insufficient nutrient intake, secondary complications, mixed parasite infection, and other coexisting diseases. Furthermore, schistosomiasis may result in loss of weight gain and weak acquired immunity [22]. As a result, animals with poor body condition and weakened immune status become more suppressed, leading to a delayed response to *Schistosoma* infection [36]. This delay provides a favorable time for the establishment and fertility of the parasites in animal bodies.

Management practice of animals was significantly associated with the prevalence of schistosomiasis. The prevalence of cattle schistosomiasis was higher in animals managed under an extensive system. This finding was comparable to other previous studies [13, 30]. This was because cattle managed in an extensive system might be more exposed to *Schistosoma bovis* compared to semi‐intensive and intensive‐reared cattle, because the disease transmission needs animals to come into contact with swamp snails and cercariae [30].

The survey result indicated that around one‐fourth of the respondents (27.5%) isolated diseased animals from healthy animals in their farms. This finding was essentially lower than a study in Pakistan [27], which reported that 71.8% of animal farmers isolated diseased animals from healthy animals. Isolation of sick animals is one important component of breaking the transmission cycle, reducing contamination of water, and decreasing human exposure to the diseases. Nearly one‐half (48.3%) of the respondents also shared the same house with animals. This report was higher (37.5%) than a study conducted in Pakistan [27]. Animal farmers practiced this risky behavior (sharing a common house) with animals that may elevate the risk of zoonosis disease transmission due to various biological, environmental, and social factors.

Less than one‐half (45.8%) of the study participants practiced reducing snails from their animal feeding and watering troughs. This result was comparable to a study done in Pakistan (41.8%) [27]. By monitoring and removing snails from animal care facilities and locations, as well as by cleaning drinking and feeding troughs, animal slaughtering areas, and animal birthing sites, physical control methods are required to lower snail populations through environmental management. In the current study, the majority (90.8%) of respondents had dewormed their animals with anthelmintics. Usually, chemical control involves the use of a synthetic or natural chemical molluscicide, and this is one of the most effective ways to control snails [38]. Although the potential biological control of freshwater snails has drawn interest due to its potential advantages for both humans and the environment when successful, improper management could have detrimental impacts on human health [39], due to various drug resistances to currently available anthelmintic medications and the clinical unavailability of viable vaccinations for snail‐borne parasitic diseases, this strategy of regulating host snail populations to interrupt the cycle of disease transmission is a substitute for minimizing the spread of such diseases [40].

All respondents (100%) reported the presence of a latrine facility in their house; however, around two‐thirds (64.2%) of the study participants had practiced defecation openly in the bush, in an open place, or in and around the water. This report was comparable with studies conducted in Addiremet town [41] which reported that 56.5% of the respondents defecated around the river and 62.5% in the bush in western Uganda [42].

More than three‐fourths (80.8%) of the study participants practiced consuming raw fish meat and vegetables. The majority of respondents also had frequent contact with open fresh water or lakes. A study conducted in Ethiopia revealed that 81.0% and 53.3% of participants, respectively, reported that eating raw meat or vegetables was the way fascioliasis was spread. About 24.0% of respondents thought that bats could spread the illness to people, but none of them knew that snails may also spread fascioliasis [43]. This condition is a bad practice that exposes hosts to trematode infection. Since snails are the sole intermediate host in the life cycle of snail‐borne parasitic diseases, such as schistosomiasis and fasciolosis, they infect them by entering the parasites′ larval stage, miracidium, and reproducing asexually within them. Thousands of cercariae are consequently released into the water, infecting the animals that come into contact with the tainted water [44].

Around one‐third (34.2%) of the study participants did swim or wash their body in a pond, river, deep well, stream, or lake. This report was lower than the study done in Addiremet town [41], which reported that 88.0% of respondents had swimming or bathing in the river. A study in Yemen also had a higher practice (58.8%) of swimming/bathing in the river [45]. A study in Uganda [42] also revealed that 53.3% and 27.6% of respondents had practiced bathing and swimming, respectively, in water. A report in the Philippines demonstrated that 58.45% and 83.56% of respondents had participated in avoiding contact with water bodies and regular mass treatment activities, respectively, to reduce schistosomiasis [46]. This variation could be due to the difference in the accessibility of tap water in diverse study areas. Swimming or bathing in infected water by an infected snail is a risky practice for contracting schistosomiasis. Numerous practices should be used to remove and control snails, as many risks for contracting snail‐borne infections during interactions with animals were identified during the survey, and the animal grazing and drinking areas were a major source of infection. More than half of the participants mentioned that the close association of family members with animals exacerbated this factor. Poor health facilities and a lack of veterinary hospitals in rural areas contributed to the spread of schistosomiasis and fascioliasis [27].

Regarding the overall practice level, a smaller portion of the respondents (12.5%) had total positive practice. The level of practice among respondents was statistically significant between different levels of educational status. Respondents who attended high school and above had better practice (29.4%) compared to respondents who attended elementary (17.1%) and those respondents who had not followed any formal education (5.9%). A similar study was reported in Pakistan [27], even though the association was not significant. Positive practices such as sanitation, hygiene, appropriate animal management, and community education are very important to reduce the prevalence of zoonotic trematodal disease. By implementing these suggested strategies, communities can protect both animal and public health, eventually leading to better human health outcomes and reduced economic burdens associated with these diseases.

## 5. Limitations of the Study

It is impossible to determine the cause‐and‐effect relationship between cattle schistosomiasis and independent variables due to the cross‐sectional nature of the study design. Since the sedimentation technique has limited sensitivity, particularly in cases of early infection and in asymptomatic patients, the actual prevalence of the disease may be higher than this figure. For the practice survey, the trustworthiness and memorization abilities of individual participants were not assessed because there were no standardized tools, even though a pilot investigation was conducted among 10 respondents and efforts were made to ensure that the respondents internalized the questions before responding.

## 6. Conclusion

In Maya City, the prevalence of schistosomiasis in cattle was high. Local breed, poor and medium body condition, older age, and extensive management were associated with the prevalence of cattle schistosomiasis. The level of zoonotic trematode prevention and control practice among cattle farmers were extremely low, and the respondents′ practice was associated with their educational attainment but not subcity, gender, age group, marital status, farming experience, and monthly income. Schistosomiasis poses a serious risk to the animals and public health sectors in Maya City, so it is crucial to improve behavioral changes related to the management practice of animals. Avoiding defecation in water bodies and contact with contaminated water are also crucial to mitigate the infection. In addition, to determine the dynamics of disease transmission in Eastern Ethiopia, it is necessary to conduct comprehensive epidemiological surveys in conjunction with the one health approach to detect species hybridization of zoonotic trematodes in the study area.

## Ethics Statement

Haramaya University, College of Veterinary Medicine Research, and the ethical review committee granted the study ethical approval (Ref. No. CVM/418/2023).

## Conflicts of Interest

The author declares no conflicts of interest.

## Funding

No funding was received for this manuscript.

## Data Availability

The data is obtained on reasonable request from the author.
